# Bridging the Allergy–Radiology Gap in Radiocontrast Media Hypersensitivity: A Patient-Level Linkage Study on Diagnostic Stewardship and Re-Exposure Outcomes

**DOI:** 10.3390/life16101715

**Published:** 2026-10-14

**Authors:** Meryem İrem Toksoy Şentürk, Ceyda Tunakan Dalgıç, Kasım Okan, Ragıp Fatih Kural, Züleyha Galata, Kutay Kırdök, Eda Aslan, Türkan Dizdar Canbaz, Ümitcan Ateş, Ecem Ay, Seda Karaaslan Yetemen, Reyhan Gümüşburun, Emine Nihal Mete Gökmen, Aytül Zerrin Sin

**Affiliations:** Division of Immunology and Allergy, Department of Internal Medicine, Faculty of Medicine, Ege University, 35100 İzmir, Türkiye; irem.toksoy@yahoo.com (M.İ.T.Ş.); kasimokan55@gmail.com (K.O.); ragipfatihkural@gmail.com (R.F.K.); zuleyhagalata61@gmail.com (Z.G.); kirdokkutay@gmail.com (K.K.); edaarslan_91@hotmail.com (E.A.); turkan88dizdar@gmail.com (T.D.C.); umitcanates@gmail.com (Ü.A.); ecem.ay@live.com (E.A.); seda.karaaslan95@gmail.com (S.K.Y.); reyhangumusburun@gmail.com (R.G.); enihalmete@yahoo.com.tr (E.N.M.G.); aytulsin@yahoo.com (A.Z.S.)

**Keywords:** contrast media hypersensitivity, iodinated contrast media, gadolinium-based contrast agents, skin testing, contrast-enhanced imaging, subsequent administration, premedication, retrospective cohort

## Abstract

**Background/Objectives**: We examined how allergy findings related to candidate-agent identification, subsequent real-world imaging and clinical outcomes. **Methods**: This single-centre retrospective cohort included 29 adults evaluated between January 2019 and May 2025 for contrast media hypersensitivity. Imaging and outcomes between allergy evaluation and a structured telephone interview in July 2026 were assessed retrospectively and verified in national electronic health records, without prospective follow-up. Primary outcomes were skin test positivity and new hypersensitivity reactions during subsequent contrast administration. **Results**: The culprit contrast agent was not documented by name in 18 patients (62.1%); median evaluation delay was 60 months. Skin tests were positive in 1/20 patients (5.0%); skin-test-negative candidate agents were identified in 19/20 (95.0%). New reactions occurred in 3/14 contrast recipients (21.4%), all with immediate Brown Grade III index reactions to iohexol. Subsequent reactions included one cardiac arrest after iohexol and two Grade II reactions after iodixanol. Of seven patients imaged without contrast, four had candidate agents but reported continuing patient and physician concerns. No Holm-adjusted comparison was significant. **Conclusions**: Skin testing identified a candidate agent in most tested patients, but candidate identification did not establish clinical tolerance or independent protection. A shared, individualised imaging plan should communicate agent selection, premedication decisions, test limitations and precautions to patients and radiologists while preserving access to necessary imaging.

## 1. Introduction

Iodinated contrast media (ICM) and gadolinium-based contrast agents (GBCA) are used in imaging for diagnosis and disease monitoring. Hypersensitivity reactions associated with these agents may occur immediately or after a delay. Immediate reactions range from mild skin manifestations to life-threatening anaphylaxis, whereas non-immediate reactions predominantly involve the skin. With the introduction of low- and iso-osmolar ICM, the frequency of immediate hypersensitivity reactions has decreased. Large series have reported immediate hypersensitivity reaction rates of 0.15–0.69% for ICM administrations and 0.06–0.17% for GBCA administrations [1].

In patients with a history of a contrast-associated reaction, planning subsequent imaging requires assessment of both the diagnostic need and the risk of a recurrent reaction. The clinical features and severity of the previous reaction and the specific agent administered are central to this decision [2]. The clinical question is therefore not limited to which contrast agent should be selected. Whether contrast is necessary, whether an appropriate imaging alternative is available and which precautions are required if contrast is administered should also be considered [3].

The European Academy of Allergy and Clinical Immunology recommends skin testing as the first diagnostic step in the evaluation of immediate and non-immediate hypersensitivity reactions to ICM. When known, the culprit agent and alternative agents should be included in the testing panel, and skin prick testing (SPT) and intradermal testing (IDT) should preferably be performed within 2–6 months of the reaction [4]. Because a negative skin test does not independently establish clinical tolerance, drug provocation testing (DPT) with a skin-test-negative agent may be considered in selected patients after assessment of the risks and benefits [2,4].

The possibility of cross-reactivity among contrast agents requires molecular structure to be considered when selecting an alternative [2,5]. Non-ionic ICM can be grouped according to the structural characteristics of their carbamoyl side chains. Group A includes iohexol, iomeprol, ioversol and iodixanol; Group B includes iobitridol; Group C includes iopromide; and Group D includes iopamidol [5]. GBCA are classified as linear or macrocyclic, but information on cross-reactivity among these agents is more limited [2,6]. Structural characteristics should therefore be considered together with skin test results, and selection of a different molecule or structural group should not be regarded as a guarantee of reaction-free administration [2,4].

The need for agent substitution, referral for allergy evaluation and premedication varies according to the characteristics of the previous reaction. The 2025 ACR/AAAAI consensus statement recommends agent substitution whenever feasible after a mild immediate ICM reaction but does not recommend premedication. After a severe immediate reaction, appropriate alternative imaging should first be considered. If use of the same contrast class is necessary, premedication should be considered together with agent substitution when possible and administration under appropriate hospital conditions [3]. The 2025 ESUR guideline also does not recommend routine premedication, although it allows a role in selected emergency situations. Premedication should therefore be considered together with appropriate agent selection and administration precautions rather than as a stand-alone protective measure [2,3].

Incomplete information about the previous reaction is an important challenge in evaluating contrast media hypersensitivity. ESUR notes that details of previous reactions are frequently unavailable or inadequately documented and recommends identifying the specific agent and including it in the testing panel when known. Skin test sensitivity may also vary according to the timing of evaluation [2]. An unknown culprit molecule does not entirely prevent evaluation with other agents, but it limits the ability to determine the relationship between the tested agent and the molecule used during the index reaction. Test results should therefore be interpreted according to the agents evaluated and the available clinical information.

Previous studies have evaluated skin-test-guided agent selection and the outcomes of subsequent administrations [7]. However, identifying an agent as a candidate for future use, administering that agent and completing the procedure without a reaction are distinct outcomes. This distinction is particularly important when culprit-agent information is missing, when the interval to evaluation is long or when testing panels differ among patients. Linking allergy evaluation records with subsequent imaging information at the patient level allows test results to be examined together with their implementation in clinical practice.

We therefore aimed to examine the contribution of allergy evaluation to identifying candidate agents for future use together with subsequent real-world imaging procedures and clinical outcomes. In patients with a history of contrast media hypersensitivity, we linked culprit-agent documentation and skin test findings with subsequent imaging choices, administered contrast agents and clinical outcomes to evaluate the pathway from allergy assessment to clinical application.

## 2. Materials and Methods

### 2.1. Study Design and Setting

This single-centre descriptive retrospective cohort study was conducted at the Adult Allergy and Clinical Immunology Unit of Ege University Faculty of Medicine. We reviewed clinical and allergy evaluation records of adults assessed between January 2019 and May 2025 for a history of ICM- or GBCA-associated hypersensitivity.

A single structured telephone interview was conducted in July 2026. The interview covered imaging procedures, contrast administrations and clinical outcomes from completion of the allergy evaluation to the interview date. Hospital and national electronic health records were also reviewed, and information was linked at the patient level. Patients were not prospectively followed after evaluation. Subsequent contrast administrations occurred during routine clinical imaging and were not provocation tests planned as part of the study.

The study was approved by the Medical Research Ethics Committee of Ege University Faculty of Medicine (Decision No. 2026-145826-7T/92; 2 July 2026). Interviews were conducted after approval. Verbal informed consent was obtained from patients and from the family member interviewed for the deceased patient. Consent was documented in the research file. Written consent could not be obtained because contact was by telephone only. The study complied with the Declaration of Helsinki.

### 2.2. Patients

**Figure 1:**
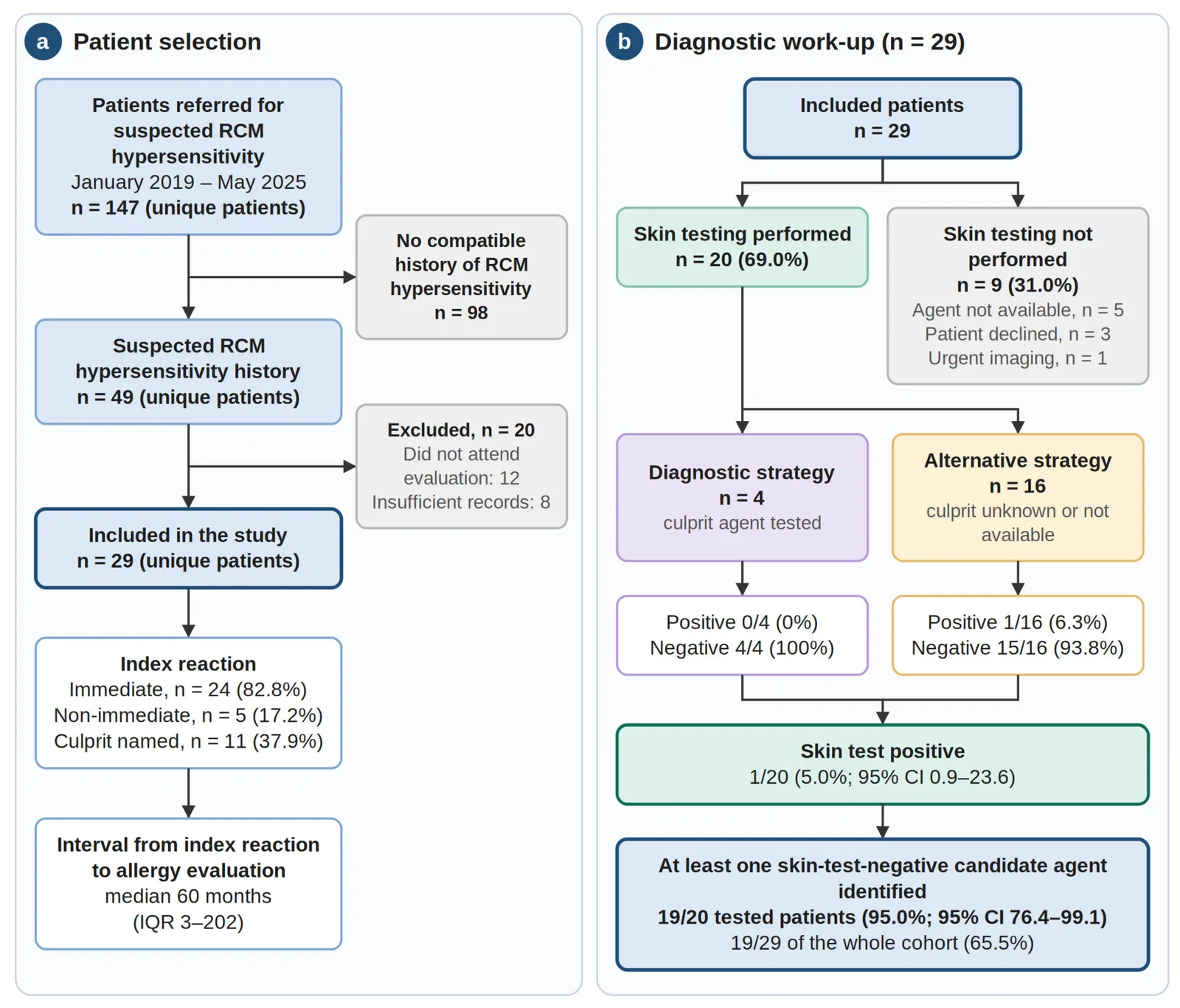
(**a**) Patient selection; (**b**) diagnostic work-up. Numbers refer to unique patients. Abbreviations: CI, confidence interval; IQR, interquartile range; RCM, radiocontrast media.

During the study period, 147 patients referred to our unit for suspected contrast media hypersensitivity were assessed. A compatible history was not identified in 98 patients. Of the remaining 49, 12 did not attend the allergy evaluation and 8 had insufficient medical records; these 20 patients were excluded. The final cohort included 29 patients (Figure 1). Each patient was counted once, regardless of repeat visits.

Eligible patients were aged 18 years or older at allergy evaluation and had completed their clinical allergy evaluation at our centre. A clinically documented reaction temporally associated with contrast administration and compatible with hypersensitivity was required. Skin testing and documentation of the specific culprit agent were not required. A long interval between the index reaction and evaluation did not preclude inclusion.

Patients were excluded if a concurrently administered drug was considered a more likely culprit or if symptoms indicated a non-allergic physiological reaction. Examples included isolated nausea or transient warmth without accompanying features suggestive of hypersensitivity.

### 2.3. Clinical Data and Index Reaction

A predefined form was used to record age, sex, atopic history and comorbidities from clinical records. Index-reaction data included contrast class, agent name when available and previous exposure to the same molecule. Time from administration to the first symptom, organ involvement and acute treatment were also recorded. Adrenaline use, hospital admission and intubation were recorded separately.

Index-reaction details were obtained from the clinical history and referral discharge reports. Atopic history was assessed using the clinical history, national electronic records and investigations performed at our centre.

Reactions were classified as immediate when the first symptom developed within 1 h of contrast administration and as non-immediate when it developed later. The interval between the index reaction and allergy evaluation was recorded in months.

Immediate reactions were graded using the Brown classification [8]. Grade I reactions were limited to the skin and subcutaneous tissue. Grade II comprised moderate reactions with respiratory, cardiovascular or gastrointestinal involvement. Grade III reactions involved hypoxia, hypotension or neurological compromise. Brown grades were not assigned to non-immediate reactions.

Available results for total serum IgE, baseline serum tryptase and absolute eosinophil count obtained during the allergy evaluation were recorded. IgE and tryptase were measured using the ImmunoCAP system (Thermo Fisher Scientific, Uppsala, Sweden). The laboratory reference limit for tryptase was <11.4 µg/L. No acute tryptase result was available from the index reaction.

### 2.4. Diagnostic Evaluation and Skin Testing

Skin tests were performed by an experienced allergy team in a setting with emergency treatment and resuscitation facilities. ICM testing followed EAACI/ENDA practice parameters [4]. For GBCA, undiluted SPT and 1:10 IDT were based on published practice [9,10]. This approach was not regarded as a validated EAACI/ENDA protocol for GBCA. The irritant potential of GBCA preparations was not separately assessed in healthy controls.

SPT was performed on the volar forearm with undiluted commercial contrast media preparations available at the time of evaluation; manufacturers varied during the study period. Concentrations were 300–320 mg iodine/mL for ICM and 0.5–1.0 mmol/mL for GBCA. Histamine 10 mg/mL (ApiPrick; ASAC Pharmaceutical, Alicante, Spain) was the positive control, and hospital-supplied sterile 0.9% sodium chloride was the negative control. Tests were read at 15–20 min. A wheal at least 3 mm larger than the negative control was considered positive.

If SPT was negative, IDT began at a 1:100 dilution as an additional safety step. Testing proceeded to 1:10 if this dilution was negative. Approximately 0.03 mL was injected to produce an initial wheal of 3–5 mm. At 15–20 min, an increase of at least 3 mm from the initial wheal with surrounding erythema was considered positive.

In patients with non-immediate reaction histories, IDT at a 1:10 dilution was read at 48 and 72 h. An infiltrated erythematous papule or induration was considered positive. Patch tests prepared in our clinic with undiluted preparations were applied to the upper back in occlusive chambers. Chambers were removed at 48 h, and tests were read at 48 and 72 h.

Skin testing used the agents available at evaluation. Evaluations were classified as “diagnostic” if a known culprit agent was tested. They were classified as “alternative” if the culprit was unknown or was known but not tested.

Patients were advised to avoid agents with positive skin tests. Agents with negative skin tests were considered candidates for future imaging, but a negative result alone did not establish clinical tolerance.

No patient underwent DPT. Reasons included safety concerns related to cardiovascular and oncological comorbidities and patients’ reluctance to receive contrast again. The absence of an agreed standard protocol for intravenous contrast provocation also influenced this decision [11].

Skin testing was not performed in 9 patients. Reasons included unavailability of a suitable agent, patient refusal and urgent imaging needs. Recommendations were based on clinical history and available agent information.

### 2.5. Ascertainment of Clinical Outcomes After Allergy Evaluation

Telephone interview questions covered contrast use, the contrast class administered and whether premedication had been given. Patients were also asked whether a reaction had occurred during or after imaging. For reported reactions, onset, clinical features, severity and treatment were recorded. Patients who could not be reached after 3 calls on separate days were classified as not contactable.

Each of the 14 patients who received contrast after evaluation underwent a single contrast-enhanced imaging procedure. Procedure completion and the administered agent were verified in national electronic health records. The post-procedure status of the 11 patients who reported no reaction was also checked in electronic records. Deaths were checked in clinical records and, when possible, with family members.

Antihistamine and corticosteroid prescriptions were verified in prescription records. However, administered doses, timing and complete regimens could not be reliably determined. Premedication was not standardised as part of the study, so its effectiveness was not analysed as a separate outcome.

Patients’ concerns about contrast use and their explanations of physicians’ imaging choices were recorded. Information on physicians’ attitudes was based on patient report.

### 2.6. Outcome Definitions

The diagnostic pathway was evaluated in all 29 patients. Availability of culprit-agent information and whether skin testing was performed were assessed. Analyses using Brown grades were restricted to the 24 immediate reactions, and the 5 non-immediate reactions were described separately. Each outcome was reported with the relevant patient group and denominator.

The primary diagnostic outcome was the proportion of tested patients with at least 1 positive skin test. This was calculated overall and separately for immediate and non-immediate reaction histories. Skin testing was also classified as diagnostic or alternative. We also calculated the proportion with at least 1 skin-test-negative candidate agent for future imaging, both among tested patients and in the full cohort.

Among patients who received contrast after allergy evaluation, the class previously tested was compared with the class administered (ICM or GBCA). Patients were class-concordant if at least 1 agent from the administered class had been tested. They were class-discordant if only the other class had been tested, and untested if no skin test had been performed.

Class concordance did not imply that the administered molecule itself had been tested or that the test result was negative. The patient with a positive test who subsequently received another agent from the same class was therefore included in the class-concordant group.

The primary clinical outcome was a new hypersensitivity reaction during contrast administration after allergy evaluation, regardless of premedication. The reaction rate was calculated only among patients who received contrast again and had a known outcome. Patients without subsequent contrast administration or with unknown outcomes were not included in the no-reaction group.

Secondary clinical outcomes included the proportion undergoing subsequent imaging, imaging type and contrast class. Premedication use was expressed as a proportion of patients receiving contrast. The association between index severity and subsequent reactions was examined only in patients with an immediate index reaction.

### 2.7. Statistical Analysis

Reporting followed the STROBE recommendations [12]. The completed checklist is provided in Supplementary Table S2. No a priori sample size calculation was performed. Analyses included eligible patients evaluated during the study period. Continuous variable distributions were assessed with the Shapiro–Wilk test. Values are presented as mean ± standard deviation or median with interquartile range, as appropriate. Categorical variables are reported as counts and percentages. Wilson 95% confidence intervals were calculated for the principal proportions.

Continuous variables were compared using the Mann–Whitney U test and categorical variables using Fisher’s exact test. Exact two-sided *p* values were used because of the small group sizes; for the Mann–Whitney U test, *p* values were obtained by complete enumeration of the permutation distribution of the rank sum, which accounts for tied values, and were reported as twice the smaller one-sided probability. Primary outcomes were summarised as counts, proportions and 95% confidence intervals.

Holm adjustment was applied to the unrounded *p* values of 6 secondary or exploratory univariable comparisons to control the family-wise Type I error. The association between class concordance and new reactions was examined overall and in the subgroup subsequently given ICM. The association between index reaction severity and new reactions was also assessed. The reaction-to-evaluation interval was examined in 2 comparisons, by class concordance and by availability of the index-agent name. The final comparison examined baseline tryptase by index reaction severity. Both unadjusted and Holm-adjusted *p* values are reported.

Firth penalised logistic regression models were used as sensitivity analyses to assess confounding. They were not treated as independent hypothesis tests and were excluded from the Holm family.

Index reaction severity and the reaction-to-evaluation interval were assessed as potential confounders. The interval was compared by class concordance and by availability of the index-agent name using the Mann–Whitney U test. Sensitivity, specificity and negative predictive value were not calculated because no standard reference assessment was available.

Firth penalised logistic regression included the 13 patients with an immediate index reaction who subsequently received contrast [13]. It examined the association between class concordance and a new hypersensitivity reaction. Patients without previous skin testing to an agent from the subsequently administered class formed the reference group.

The first model included class concordance and index severity (Grade III or Grade I–II). The sensitivity model also included the log_2_-transformed reaction-to-evaluation interval in months. Results are reported as adjusted odds ratios, 95% profile penalised-likelihood confidence intervals and penalised likelihood-ratio test *p* values.

Descriptive analyses were performed using IBM SPSS Statistics version 27.0 (IBM Corp., Armonk, NY, USA). Exact permutation *p* values, Holm adjustment and Firth penalised logistic regression models were computed in Python 3.12 (Python Software Foundation, Wilmington, DE, USA) using SciPy 1.17 (SciPy community; https://scipy.org). Estimation was based on penalised maximum likelihood with a Jeffreys-prior penalty.

### 2.8. Use of Generative Artificial Intelligence

Generative artificial intelligence tools, Claude Fable 5.1 (Anthropic, San Francisco, CA, USA) and ChatGPT-6 Astra (OpenAI, San Francisco, CA, USA), were used to assist with language editing, with the preparation of the figures and the graphical abstract from the authors’ data, and with the implementation of the statistical code for the Firth penalised logistic regression, which the authors verified against the source data. These tools were not used to generate, collect or interpret data. The authors reviewed and edited all outputs and take full responsibility for the content.

## 3. Results

### 3.1. Study Population and Index Reactions

The study included 29 adults evaluated because of a history of immediate or non-immediate hypersensitivity associated with contrast media (Figure 1). The mean age was 57.4 ± 11.2 years, and 22 patients (75.9%) were women. Hypertension was present in 18 patients (62.1%), cardiovascular disease in 13 (44.8%) and malignancy in eight (27.6%). In addition, 15 patients (51.7%) had an allergic disease, and 14 (48.3%) had a history of allergy to drugs other than contrast media. Demographic and clinical characteristics are presented in Table 1.

The culprit contrast medium was an ICM in 22 patients (75.9%) and a GBCA in seven (24.1%); the specific culprit agent was documented by name in 11 patients (37.9%). Among named agents, iohexol was most frequently reported and accounted for 9 of 22 patients in the ICM group (40.9%). The detailed agent distribution is presented in Table 2.

Index reactions were classified as immediate in 24 patients (82.8%) and non-immediate in five (17.2%). Among the 24 patients with an immediate reaction, 11 (45.8%) were classified as Brown Grade III, 10 (41.7%) as Grade II and three (12.5%) as Grade I.

Most immediate reactions involved more than one organ system. Respiratory involvement (18/24; 75.0%) and skin involvement (17/24; 70.8%) were most common. Adrenaline was documented in five patients (20.8%), two were admitted to hospital and one was intubated; no cardiac or respiratory arrest occurred at the index reaction.

All five non-immediate reactions were self-limited and presented as an erythematous maculopapular exanthema, with generalised pruritus in three (60.0%); urticaria and angioedema were not recorded, and none of these patients was admitted to hospital. Patient-level data on index reactions, allergy evaluations, subsequent imaging and clinical outcomes are presented in Supplementary Table S1.

### 3.2. Culprit-Agent Information and Reaction-to-Evaluation Interval

For 18 patients (62.1%), the name of the contrast agent used during the index reaction could not be retrieved from the available records. Skin testing was performed in 8 of the 11 patients with a known agent and in 12 of the 18 patients whose agent name could not be retrieved. In patients with an unknown agent, it could not be confirmed whether the tested agent was structurally different from the culprit agent.

The median interval between the index reaction and the allergy evaluation was 60 months (IQR 3–202), and the interval exceeded 120 months in nine patients. The median interval was 4 months (range 1–202) in the 11 patients whose index-agent name was known and 78 months (range 1–516) in the 18 patients whose agent name could not be retrieved (Mann–Whitney U test, unadjusted *p* = 0.030; Holm-adjusted *p* = 0.114).

### 3.3. Skin Test Results and Identification of Candidate Agents for Future Use

Skin testing was performed in 20 of the 29 patients (69.0%). The four patients whose testing panel included the known culprit agent were included in the diagnostic evaluation group, and the other 16 in the alternative evaluation group. The remaining nine patients (31.0%) did not undergo skin testing.

An IDT performed with gadobutrol at a 1:10 dilution was positive in one patient whose index reaction was immediate and Grade II. Gadobutrol was therefore not recommended for future imaging. The positivity rate was 1/20 among all tested patients (5.0%; 95% CI 0.9–23.6) and 1/16 among those with an immediate reaction history (6.3%; 95% CI 1.1–28.3). Delayed IDT readings and patch tests were negative in all four patients with non-immediate index reactions [0/4 (0%; 95% CI 0–49.0)]. All tests performed with known culprit agents and all ICM tests were negative.

Among the 20 tested patients, at least one skin-test-negative agent was identified as a candidate for future imaging in 19 [19/20 (95.0%; 95% CI 76.4–99.1)]. These patients represented 65.5% of the full cohort (19/29).

### 3.4. Imaging After Allergy Evaluation and New Hypersensitivity Reactions

**Figure 2:**
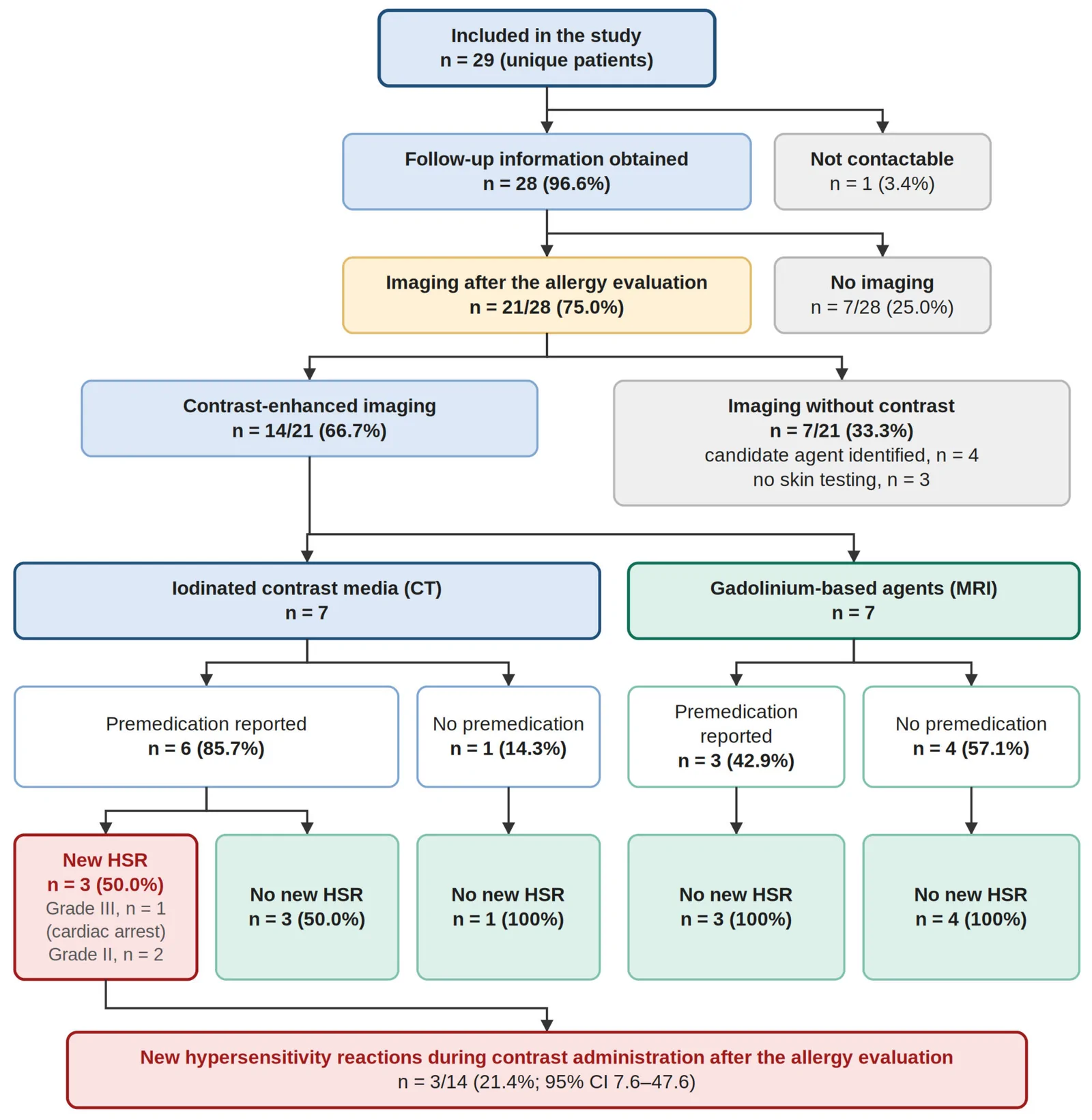
Imaging and contrast administration after the allergy evaluation, and clinical outcomes. Abbreviations: CI, confidence interval; CT, computed tomography; HSR, hypersensitivity reaction; MRI, magnetic resonance imaging.

Post-evaluation imaging information was available for 28 patients (96.6%); one could not be reached after three calls. Of the 28 patients, 21 underwent imaging, with contrast in 14 (7 ICM, 7 GBCA) and without contrast in seven (Figure 2). Premedication was reported for 9/14 contrast administrations (64.3%; 6 ICM, 3 GBCA). Of the seven patients imaged without contrast, four had a previously identified skin-test-negative candidate agent and three had not undergone skin testing. The median interval from the allergy evaluation to the telephone interview was 42 months.

Patients who underwent imaging without contrast despite having undergone skin testing reported continuing concerns about contrast use and stated that their physicians had also requested non-contrast examinations because of concern about a reaction.

Among the 14 patients who received contrast again, 13 had an immediate and one had a non-immediate index reaction. New hypersensitivity reactions developed in three patients [3/14 (21.4%; 95% CI 7.6–47.6)]. All three had a history of an immediate Brown Grade III index reaction associated with iohexol [3/5 (60.0%) vs. 0/8 (0%); Fisher’s exact test, unadjusted *p* = 0.035; Holm-adjusted *p* = 0.114], and all new reactions occurred during ICM administration at external centres under reported premedication.

One patient developed cardiac arrest after readministration of iohexol and required intensive care admission. The other two patients developed Grade II reactions after iodixanol (Table 3). The patient who had a cardiac arrest survived; 1 of the 3 patients later died of her underlying malignancy (Table 3), the only death in the cohort. No reactions were reported after GBCA administration. The patient with a positive gadobutrol skin test underwent contrast-enhanced MR imaging with gadoterate meglumine under premedication and did not develop a reaction.

**Figure 3:**
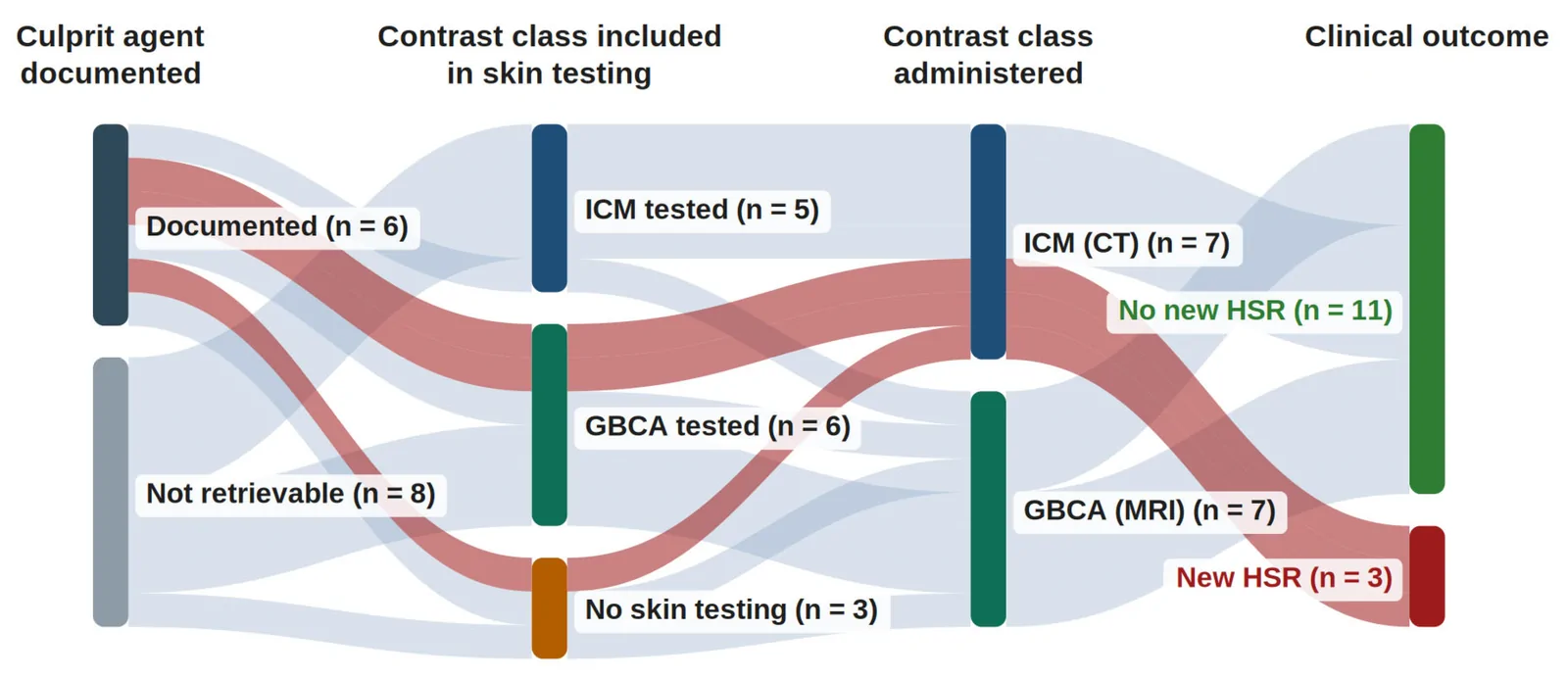
Patient-level linkage between documentation of the culprit agent, the contrast class included in skin testing, the contrast class administered after the allergy evaluation and the clinical outcome in the 14 patients who received contrast again. Each ribbon is one patient; red ribbons, new hypersensitivity reaction. Abbreviations: CT, computed tomography; GBCA, gadolinium-based contrast agent; HSR, hypersensitivity reaction; ICM, iodinated contrast media; MRI, magnetic resonance imaging.

For the 14 patients who received contrast again, the contrast class evaluated in previous skin testing was compared with the class used in the subsequent imaging procedure. Eight patients were class-concordant, three were class-discordant and three had not undergone skin testing (Figure 3). No reaction occurred in the eight patients previously tested with an agent from the class subsequently administered, whereas new hypersensitivity reactions occurred in 3 of the 6 patients who had not been tested with the subsequently administered class [0/8 (0%; 95% CI 0–32.4) vs. 3/6 (50.0%; 95% CI 18.8–81.2); Fisher’s exact test, unadjusted *p* = 0.055; Holm-adjusted *p* = 0.114].

The interval between the index reaction and allergy evaluation was longer in class-concordant patients [median 240 months (range 4–516) vs. 12 months (range 1–66); Mann–Whitney U test, unadjusted *p* = 0.012; Holm-adjusted *p* = 0.072]. The association between class concordance and a new hypersensitivity reaction was evaluated using Firth penalised logistic regression adjusted for index reaction severity. Among these 13 patients, seven were class-concordant (0 reactions) and six were class-discordant or untested (three reactions). In the first model, the adjusted OR was 0.16 for class concordance (95% CI 0.001–4.02; *p* = 0.264) and 10.03 for a Grade III index reaction (95% CI 0.53–1438; *p* = 0.127). In the sensitivity model that additionally included the log_2_-transformed reaction-to-evaluation interval, the adjusted OR was 0.19 for class concordance (95% CI 0.0014–4.06; *p* = 0.281), 8.69 for a Grade III index reaction (95% CI 0.28–2143; *p* = 0.226) and 1.01 for each 2-fold increase in the evaluation interval (95% CI 0.47–2.48; *p* = 0.972). Confidence intervals were wide in both models, and the independent effects could not be reliably distinguished.

In the exploratory subgroup analysis of the seven patients who received ICM again, no reaction occurred among those previously tested with ICM, whereas all patients without prior ICM testing developed a new hypersensitivity reaction [0/4 (0%; 95% CI 0–49.0) vs. 3/3 (100%; 95% CI 43.9–100); Fisher’s exact test, unadjusted *p* = 0.029; Holm-adjusted *p* = 0.114]. All three patients without prior ICM testing had a Grade III index reaction, whereas the four patients with prior ICM testing had Grade I–II index reactions. The effects of skin testing and index reaction severity on the difference in reaction rates could therefore not be separated in this subgroup.

### 3.5. Baseline Serum Tryptase

Baseline serum tryptase was measured in 10 patients (34.5%), and all values were within the laboratory reference range (observed range 3.03–8.46 µg/L). One patient with a non-immediate index reaction was excluded from the severity analysis. Among the nine patients with immediate reactions, baseline tryptase was higher in those with a Grade III index reaction than in those with a Grade I–II reaction [median 8.14 µg/L (range 6.97–8.46), *n* = 4 vs. 3.79 µg/L (range 3.03–6.50), *n* = 5; Mann–Whitney U test, unadjusted *p* = 0.016; Holm-adjusted *p* = 0.079]. None of the six exploratory univariable comparisons retained *p* < 0.05 after Holm adjustment.

Acute tryptase measurements obtained during the index reaction were unavailable for every patient. Baseline tryptase had not been measured in any of the three patients who developed a new hypersensitivity reaction. Its association with the development of a subsequent reaction could therefore not be evaluated.

## 4. Discussion

This study linked allergy assessment for contrast media hypersensitivity with subsequent imaging decisions and clinical outcomes at the patient level. It included patients with an unknown culprit agent, a long reaction-to-evaluation interval or no skin testing, reflecting the varied conditions encountered in routine care. Its main contribution was to examine how skin test findings related to subsequent agent selection, premedication and outcomes.

Missing culprit-agent information limited both the evaluation of the suspected agent and the selection of alternatives based on structural characteristics. Current guidelines emphasise the need for agent-specific and reaction-specific documentation. The 2025 ESUR guideline recommends recording the agent’s name and dose, reaction onset, type, severity, clinical features and treatment [1]. The 2025 ACR/AAAAI consensus statement similarly recommends documenting the specific agent and reaction details in the electronic health record and correcting incomplete or inaccurate entries when new information becomes available [3]. A general “contrast allergy” label is therefore insufficient. Information about the agent, the reaction and any allergy assessment should be accessible to the radiologist planning subsequent imaging. Agent selection and precautions should reflect the patient’s clinical needs, with input from an allergist when appropriate [2,3].

Missing agent information should be distinguished from uncertainty about the reaction mechanism. In our cohort, the inability to identify the culprit agent mainly reflected incomplete previous records. It did not, by itself, indicate a non-allergic mechanism. A negative skin test likewise does not establish an IgE-independent or non-allergic mechanism. Our retrospective study could not determine the immunological mechanisms underlying the reactions.

Skin test positivity depends on the patient population, the agents tested and the timing of testing. Yoon et al. reported positivity in 17% of patients with immediate ICM reactions and 52% of those with severe immediate reactions in their meta-analysis [14]. Our cohort included both immediate and non-immediate reactions to ICM and GBCA, and the known culprit agent was tested in only a few patients. These differences limit direct comparison of positivity rates. Testing delays may also have contributed to our low positivity rate. In their multicentre study, Brockow et al. reported higher positivity when patients with immediate reactions were tested within 2–6 months, with lower rates after longer intervals [15]. The 2025 ESUR guideline also identifies this period as the most sensitive for skin testing and notes that sensitivity may decline over time [2]. However, we could not determine the contribution of testing delays because culprit-agent information was often missing and testing panels differed between patients.

A skin-test-negative candidate agent is not proof of clinical tolerance. Schrijvers et al. reported tolerance in 201 of 216 patients with negative skin tests who subsequently received ICM (93.1%) [16]. This proportion describes outcomes among patients who received contrast again. In our study, DPT was not performed, and not all patients with an identified candidate agent subsequently received contrast. The candidate-agent identification rate therefore cannot be interpreted as a tolerance rate. Similarly, an administration without a reaction does not establish an independent protective effect of skin testing or agent selection. Nevertheless, skin test positivity alone does not capture all the information that allergy assessment can provide for planning future contrast use.

All patients who developed a new hypersensitivity reaction had a history of a severe index reaction associated with iohexol. Of the new reactions, 2 occurred after iodixanol administration. Iohexol and iodixanol belong to the same structural group [5]. Pinyopornpanish et al. also reported recurrent reactions in 3 of 6 patients who had received both agents in their retrospective study [17]. This observation supports the possibility of recurrent reactions involving this agent pair but does not establish immunological cross-reactivity.

Kim et al. provided broader evidence on structure-based agent selection by evaluating 3800 subsequent ICM administrations in 1066 patients. Compared with readministration of the same agent, switching to an agent without a shared carbamoyl side chain was associated with a lower risk of recurrence (OR 0.51; 95% CI 0.37–0.69). Switching to a different agent with a shared side chain did not show a similar reduction (OR 0.98; 95% CI 0.64–1.50) [18]. These findings support considering structural features when selecting an alternative agent, rather than relying only on a different molecular name. However, the relevant patients in our cohort had not undergone skin testing with iohexol or iodixanol. We therefore could not directly assess the contribution of structural similarity to their reactions.

No reactions occurred in patients previously tested with an agent from the contrast class subsequently administered. However, class concordance does not mean that the administered molecule itself was tested or had a negative result. The groups also differed in index reaction severity and the reaction-to-evaluation interval. None of the exploratory univariable comparisons remained statistically significant after Holm adjustment, and the Firth models produced wide confidence intervals. These findings are therefore exploratory and do not establish an independent protective effect. In their prospective study, Lee et al. reported fewer and less severe recurrent reactions under a strategy combining skin testing, agent substitution and premedication according to index reaction severity [7]. The available evidence supports a comprehensive management approach.

Premedication was reported in all patients who developed a new reaction. However, we could not assess whether the regimens were appropriate because their doses and timing could not be confirmed. Reactions despite premedication have also been reported elsewhere [19,20]. The 2025 ESUR guideline does not recommend routine premedication because evidence that it prevents severe immediate reactions is insufficient. It considers premedication an option when emergency contrast administration is required in a patient with a severe reaction history and an unknown culprit agent [2]. The 2025 ACR/AAAAI consensus statement recommends considering alternative imaging first in patients with a severe immediate ICM reaction history. If the same contrast class is necessary, premedication should be considered alongside agent substitution when possible, with administration under appropriate clinical conditions [3]. Premedication should therefore not replace suitable agent selection or individual risk assessment. As evidence continues to evolve, decisions about agent substitution, allergy evaluation and premedication should be individualised using current guidelines, clinical judgement, imaging needs and patient-specific risks.

Experimental studies showed that iohexol and gadoterate meglumine can activate mast cells through MRGPRX2 independently of IgE [21,22]. These findings support possible non-IgE-mediated mechanisms. However, our study included no mechanistic investigations, so we could not determine whether these pathways contributed to the observed reactions.

All baseline tryptase values were within the laboratory reference range. Nevertheless, patients with a Grade III index reaction had higher levels in the unadjusted comparison. This difference did not remain statistically significant after Holm adjustment. A similar association between baseline tryptase and reaction severity was reported with other anaphylaxis triggers [23]. Measurements were available for only a small number of patients, and none of those who developed a subsequent reaction had a baseline measurement. We therefore could not assess the role of baseline tryptase in predicting recurrent reactions.

Of the seven patients imaged without contrast, four had a previously identified skin-test-negative candidate agent. These patients reported that their own concerns about contrast use and their physicians’ concerns about another reaction influenced the imaging decision. This finding suggests that identifying a candidate agent does not, by itself, ensure its use in clinical practice. The ACR/AAAAI and ESUR recommendations emphasise balancing the risk of recurrence against the potential consequences of avoiding clinically necessary imaging [2,3]. Allergy assessment should therefore lead to a shared imaging plan that identifies candidate agents, explains test limitations and specifies precautions. Communicating this plan to the patient and the physician arranging imaging, with collaboration between allergists and radiologists, may help maintain access to necessary imaging while managing reaction risk.

### Study Limitations

This single-centre retrospective study included patients referred to a tertiary allergy unit. Referral patterns and the exclusion of patients who did not attend evaluation or had insufficient records may have introduced selection bias of undetermined direction and magnitude. Differences in agent availability and clinical pathways between settings may also limit generalisability.

Of 29 patients, 14 subsequently received contrast and three developed new reactions, limiting precision. In the ICM subgroup, testing status could not be separated from index reaction severity. Firth models in the 13 contrast recipients with immediate index reactions yielded wide confidence intervals after adjustment for severity and, in the sensitivity model, evaluation delay. These comparisons remain exploratory.

Incomplete historical information may have caused misclassification of reaction type or Brown grade. The inclusion of ICM and GBCA reactions and both immediate and non-immediate phenotypes limits interpretation of pooled rates, despite separate reporting and restriction of severity analyses to immediate reactions. Only four patients with non-immediate reactions underwent testing.

The culprit agent was unknown in 18 patients and tested in only four. Missing agent information, variable panels and long evaluation delays limited interpretation of test positivity. Guidance addresses patients with unknown culprit agents [24], but older preparations could not be characterised in this cohort. ESUR recommends low- or iso-osmolar non-ionic ICM [25]; lower adverse reaction rates with low-osmolar non-ionic agents were documented in 1990 [26]. A long interval alone does not establish exposure to high-osmolar ionic agents.

DPT was not performed because of safety concerns related to comorbidities, patient reluctance and the lack of a generally accepted standardised intravenous contrast provocation protocol [11]. Without a reference assessment, skin test sensitivity, specificity and negative predictive value could not be calculated. Negative tests did not confirm clinical tolerance. Class concordance did not establish that the administered molecule had been tested or had a negative result. The 1:10 intradermal dilution of gadobutrol was consistent with published practice [9,10], but its irritant potential was not separately assessed in healthy controls in our study.

Subsequent contrast use reflected clinical needs and patient and clinician choices. Agent selection and premedication were not standardised; premedication doses and timing could not be reliably established. Their effects could not be separated from baseline risk. The observed reaction rate applies only to contrast recipients with known outcomes. These data cannot establish the safety of planned readministration or independent protection from testing or premedication.

Outcomes were assessed through a single structured telephone interview and electronic records, without prospective follow-up. Intervals between evaluation and interview varied, allowing different opportunities for imaging; the reaction rate is not a time-specific estimate of long-term cumulative risk. Although administrations and agents were verified, incomplete recall or documentation of mild reactions may have led to under-ascertainment. The unreachable patient remained in the cohort but was not classified as reaction-free. Patients’ concerns and physicians’ reasons for non-contrast imaging were patient-reported, limiting assessment of the clinical appropriateness of these decisions.

No acute tryptase measurements were available from index reactions. Baseline tryptase was available in 10 patients, but in none of the three with a subsequent reaction, preventing assessment of its predictive role. No mechanistic investigations were performed, so the pathways underlying the reactions could not be determined.

## 5. Conclusions

In this retrospective real-world cohort, at least one candidate agent for future use was identified in most patients who underwent skin testing, despite missing culprit-agent information and long reaction-to-evaluation intervals. This finding does not establish clinical tolerance. New hypersensitivity reactions occurred in 3 of the 14 patients who subsequently received contrast, all of whom had a severe index reaction history. The small sample size and confounding factors prevented reliable assessment of an independent protective effect of skin testing or class concordance.

By linking allergy assessment with subsequent imaging decisions and outcomes at the patient level, this study highlights the need to translate allergy findings into an individual imaging plan. Agent selection and premedication decisions should consider the allergy assessment, previous reaction, necessity and urgency of imaging, current guidelines and clinical judgement in this evolving area. The plan should identify candidate agents, explain test limitations and specify precautions, and should be communicated to both the patient and the radiologist. The aim is to manage recurrent reaction risk while preserving access to clinically necessary diagnostic imaging.

## Figures and Tables

**Table 1:** Demographic and clinical characteristics of the cohort (*n* = 29).

Characteristic	Value
**Demographics**	
Age, years (mean ± SD)	57.4 ± 11.2
Female/male	22 (75.9)/7 (24.1)
Interval from index reaction to allergy evaluation, months, median (IQR)	60 (3–202)
**Comorbidities**	
Hypertension	18 (62.1)
Cardiovascular disease	13 (44.8)
Psychiatric disorder	11 (37.9)
Diabetes mellitus	9 (31.0)
Malignancy	8 (27.6)
Chronic obstructive pulmonary disease	2 (6.9)
**Allergic and immunological background**	
Any allergic disease	15 (51.7)
Drug allergy unrelated to contrast media	14 (48.3)
Allergic rhinitis	10 (34.5)
Asthma	8 (27.6)
Atopic dermatitis	3 (10.3)
Venom allergy	3 (10.3)
Food allergy	2 (6.9)

**Table 2:** Index reaction characteristics and acute clinical presentation (*n* = 29).

Parameter	*n* (%)
**Culprit radiocontrast medium**	
Iodinated contrast media (ICM)	22 (75.9)
Iohexol	9/22 (40.9)
Iopromide	1/22 (4.5)
Not specified	12/22 (54.5)
Gadolinium-based contrast agents (GBCA)	7 (24.1)
Gadobutrol	1/7 (14.3)
Not specified	6/7 (85.7)
**Reaction type**	
Immediate (≤1 h)	24 (82.8)
Onset within the first 5 min	16/24 (66.7)
Non-immediate (>1 h)	5 (17.2)
Previous imaging with the same contrast agent (patient-reported)	7 (24.1)
**Severity, Brown grade (immediate reactions, *n* = 24)**	
Grade I	3 (12.5)
Grade II	10 (41.7)
Grade III	11 (45.8)
**Acute management**	
Adrenaline given	5/24 (20.8)
Hospital admission	2 (6.9)
Intubation	1 (3.4)
Cardiac or respiratory arrest at the index reaction	0 (0)
**Laboratory findings**	
Total IgE, kU/L, median (IQR) (*n* = 11)	41.6 (23.1–156.5)
Absolute eosinophil count, /µL, median (IQR) (*n* = 21)	170 (30–210)
Baseline tryptase, µg/L, range (*n* = 10)	3.03–8.46
Grade III index reaction, median (range), *n* = 4	8.14 (6.97–8.46)
Grade I–II index reaction, median (range), *n* = 5	3.79 (3.03–6.50)

Laboratory values were measured at the allergy evaluation, not during the index reaction. Abbreviations: GBCA, gadolinium-based contrast agent; ICM, iodinated contrast media; IQR, interquartile range. Percentages for severity are of the 24 immediate reactions; percentages for acute management are of the whole cohort unless stated otherwise.

**Table 3:** Clinical characteristics and outcomes of the three patients with a new hypersensitivity reaction after the allergy evaluation (*n* = 3).

Parameter	Patient 1	Patient 2	Patient 3
**Demographics and comorbidity**			
Age/sex	63/M	51/F	34/F
Comorbidity	HT, CVD, CAD, COPD	HT	Malignancy
Allergic background	None	Atopy, AR	None
Total IgE, kU/L	NM	512	NM
**Index reaction**			
Culprit agent	Iohexol	Iohexol	Iohexol
Onset	<5 min	<5 min	<5 min
Brown grade	III	III	III
Structural group (Vega classification)	Group A	Group A	Group A
**Diagnostic work-up**			
Skin testing	NP	Alternative GBCA	Alternative GBCA
Result	NA	Negative	Negative
Reason the culprit class was not tested	Alternative agent could not be obtained; imaging became urgent	Only an alternative GBCA was tested	Only an alternative GBCA was tested
Index reaction to allergy evaluation, months	1	60	3
**Subsequent contrast administration and outcome**			
Index reaction to subsequent administration, months	3	67	24
Agent given	Iohexol (culprit)	Iodixanol	Iodixanol
Modality	CT	CT	CT
Premedication	Yes	Yes	Yes
Outcome	Cardiac arrest, ICU	Grade II reaction	Grade II reaction
Vital status at interview	Alive	Alive	Died (malignancy)

Premedication as reported by the patient and verified in prescription records. Abbreviations: AR, allergic rhinitis; CAD, coronary artery disease; COPD, chronic obstructive pulmonary disease; CT, computed tomography; CVD, cardiovascular disease; GBCA, gadolinium-based contrast agent; HT, hypertension; ICU, intensive care unit; IgE, immunoglobulin E; NA, not applicable; NM, not measured; NP, not performed.

## Data Availability

The data presented in this study and the analysis code are available on reasonable request from the corresponding author. The data are not publicly available because of privacy restrictions.

## Supplementary Materials

The following supporting information can be downloaded at: https://www.mdpi.com/article/10.3390/life16101715/s1, Table S1: Anonymised patient-level linkage of index reactions, allergy evaluations, subsequent contrast administrations and clinical outcomes (*n* = 29); Table S2: STROBE Statement—checklist of items that should be included in reports of cohort studies, with the location of each item in the manuscript.

## Abbreviations

The following abbreviations are used in this manuscript:

AAAAI	American Academy of Allergy, Asthma & Immunology
ACR	American College of Radiology
CI	confidence interval
CT	computed tomography
DPT	drug provocation test
EAACI	European Academy of Allergy and Clinical Immunology
ENDA	European Network on Drug Allergy
ESUR	European Society of Urogenital Radiology
GBCA	gadolinium-based contrast agent
HSR	hypersensitivity reaction
ICM	iodinated contrast media
IDT	intradermal test
IQR	interquartile range
MRGPRX2	Mas-related G protein-coupled receptor X2
MRI	magnetic resonance imaging
OR	odds ratio
RCM	radiocontrast media
SD	standard deviation
SPT	skin prick test
STROBE	Strengthening the Reporting of Observational Studies in Epidemiology

## References

[1] van der Molen A.J., van de Ven A.A.J.M., Vega F., Dekkers I.A., Geenen R.W.F., Bellin M.-F., Bertolotto M., Brismar T.B., Clément O., Correas J.-M. (2025). Hypersensitivity reactions to contrast media: Part 1. Management of immediate and non-immediate hypersensitivity reactions in adults. Updated guidelines by the ESUR Contrast Media Safety Committee. Eur. Radiol..

[2] van der Molen A.J., van de Ven A.A.J.M., Vega F., Dekkers I.A., Geenen R.W.F., Bellin M.-F., Bertolotto M., Brismar T.B., Clément O., Correas J.-M. (2025). Hypersensitivity reactions to contrast media: Part 2. Prevention of recurrent hypersensitivity reactions in adults. Updated guidelines by the ESUR Contrast Media Safety Committee. Eur. Radiol..

[3] Wang C., Ramsey A., Lang D., Copaescu A.M., Krishnan P., Kuruvilla M., Mervak B., Newhouse J., Sumkin A., Saff R. (2025). Management and prevention of hypersensitivity reactions to radiocontrast media: A consensus statement from the American College of Radiology and the American Academy of Allergy, Asthma, & Immunology. J. Allergy Clin. Immunol. Pract..

[4] Torres M.J., Trautmann A., Böhm I., Scherer K., Barbaud A., Bavbek S., Bonadonna P., Cernadas J.R., Chiriac A.M., Gaeta F. (2021). Practice parameters for diagnosing and managing iodinated contrast media hypersensitivity. Allergy.

[5] Vega F., van de Ven A.A.J.M., van der Molen A.J. (2024). Cross-reactivity in hypersensitivity reactions to contrast agents: New classification and guide for clinical practice. Eur. Radiol..

[6] Sáenz de Santa María R., Labella M., Bogas G., Doña I., Torres M.J. (2023). Hypersensitivity to gadolinium-based contrast. Curr. Opin. Allergy Clin. Immunol..

[7] Lee J.-H., Yoo Y., Kim S.-R., Lee J.-H., Kim S.-Y., An J., Park S.-Y., Park H.-K., Kim S., Song W.-J. (2023). Skin test-guided strategy to select alternative iodinated contrast media in patients with immediate hypersensitivity reaction: A prospective confirmative study. J. Allergy Clin. Immunol. Pract..

[8] Brown S.G.A. (2004). Clinical features and severity grading of anaphylaxis. J. Allergy Clin. Immunol..

[9] Brockow K., Garvey L.H., Aberer W., Atanaskovic-Markovic M., Barbaud A., Bilo M.B., Bircher A., Blanca M., Bonadonna B., Campi P. (2013). Skin test concentrations for systemically administered drugs: An ENDA/EAACI Drug Allergy Interest Group position paper. Allergy.

[10] Grueber H.P., Helbling A., Joerg L. (2021). Skin test results and cross-reactivity patterns in IgE- and T-cell-mediated allergy to gadolinium-based contrast agents. Allergy Asthma Immunol. Res..

[11] Barbaud A., Garvey L.H., Torres M., Laguna J.J., Arcolaci A., Bonadonna P., Hofmeier K.S., Chiriac A.M., Cernadas J., Caubet J.C. (2024). EAACI/ENDA position paper on drug provocation testing. Allergy.

[12] von Elm E., Altman D.G., Egger M., Pocock S.J., Gøtzsche P.C., Vandenbroucke J.P., Initiative S. (2007). The Strengthening the Reporting of Observational Studies in Epidemiology (STROBE) statement: Guidelines for reporting observational studies. Lancet.

[13] Heinze G., Schemper M. (2002). A solution to the problem of separation in logistic regression. Stat. Med..

[14] Yoon S.H., Lee S.-Y., Kang H.-R., Kim J.-Y., Hahn S., Park C.M., Chang Y.-S., Goo J.M., Cho S.-H. (2015). Skin tests in patients with hypersensitivity reaction to iodinated contrast media: A meta-analysis. Allergy.

[15] Brockow K., Romano A., Aberer W., Bircher A.J., Barbaud A., Bonadonna P., Faria E., Kanny G., Lerch M., Pichler W.J. (2009). Skin testing in patients with hypersensitivity reactions to iodinated contrast media: A European multicenter study. Allergy.

[16] Schrijvers R., Breynaert C., Ahmedali Y., Bourrain J.L., Demoly P., Chiriac A.M. (2018). Skin testing for suspected iodinated contrast media hypersensitivity. J. Allergy Clin. Immunol. Pract..

[17] Pinyopornpanish K., Phithakham C., Prakaikietikul P., Aramrat C., Pinyopornpanish K. (2025). Characterization of hypersensitivity to iodinated contrast media: Insights from a six-year cohort of 26,465 procedures. Healthcare.

[18] Kim S., Jeon K.N., Jung J.-W., Park H.-K., Lee W., Lee J., Kang H.-R. (2023). Substitution with low-osmolar iodinated contrast agent to minimize recurrent immediate hypersensitivity reaction. Radiology.

[19] Beiner L., Jeong J., Kang D.Y., Lombardo P., Kang H.-R., Boehm I. (2024). Immediate hypersensitivity reactions to iodinated contrast media despite drug prophylaxis: A comparative retrospective cohort study of breakthrough reactions in Bern (Switzerland) and Seoul (South Korea). Quant. Imaging Med. Surg..

[20] Jingu A., Fukuda J., Taketomi-Takahashi A., Tsushima Y. (2014). Breakthrough reactions of iodinated and gadolinium contrast media after oral steroid premedication protocol. BMC Med. Imaging.

[21] Yuan F., Zhang C., Sun M., Wu D., Cheng L., Pan B., Li T., Che D. (2021). MRGPRX2 mediates immediate-type pseudo-allergic reactions induced by iodine-containing iohexol. Biomed. Pharmacother..

[22] Ruiz de Azcárate P.H., López-Sanz C., López-Raigada A., Vega F., Jiménez-Saiz R., Blanco C. (2023). Meglumine gadoterate induces immunoglobulin-independent human mast cell activation via MRGPRX2. Allergy.

[23] Aniceto V., Dias M.M., Melo J.M., Trevisan-Neto O., Aragon D.C., Maia L.S., Moreno A.S., Arruda L.K. (2019). Serum baseline tryptase level as a marker for the severity of anaphylaxis. Int. Arch. Allergy Immunol..

[24] Byrne A., Macdonald D.B., Kirkpatrick I.D.C., Pham M., Copaescu A.M., McInnes M.D.F., Ling L., Ellis A., Costa A.F. (2026). CAR/CSACI practice guidance for contrast media hypersensitivity: Update for unknown contrast agents. Can. Assoc. Radiol. J..

[25] European Society of Urogenital Radiology (2018). ESUR Guidelines on Contrast Agents.

[26] Katayama H., Yamaguchi K., Kozuka T., Takashima T., Seez P., Matsuura K. (1990). Adverse reactions to ionic and nonionic contrast media. A report from the Japanese Committee on the Safety of Contrast Media. Radiology.

