# Supplementary material for: Bridging the Allergy–Radiology Gap in Radiocontrast Media Hypersensitivity: A Patient-Level Linkage Study on Diagnostic Stewardship and Re-Exposure Outcomes
Source: Life (Basel). 2026 Oct 14;16(10):1715. doi: 10.3390/life16101715 (PMC13706518; doi:10.3390/life16101715)
Supplement: Supplementary file 1 [file life-16-01715-s001.zip › life-4614101-supplementary.pdf]

Supplementary Materials

Bridging the Allergy–Radiology Gap in Radiocontrast Media Hypersensitivity: A Patient-Level Linkage Study on Diagnostic Stewardship and Re-Exposure Outcomes

Meryem İrem Toksoy Şentürk, Ceyda Tunakan Dalgıç *, Kasım Okan, Ragıp Fatih Kural, Züleyha Galata, Kutay Kırdök, Eda Aslan, Türkan Dizdar Canbaz, Ümitcan Ateş, Ecem Ay, Seda Karaaslan Yetemen, Reyhan Gümüşburun, Emine Nihal Mete Gökmen and Aytül Zerrin Sin

Table S1. Anonymised patient-level linkage of index reactions, allergy evaluations, subsequent contrast administrations and clinical outcomes (n = 29).

Patient	Age/sex	Culprit agent	Index reaction	Time to allergy evaluation, months	Skin test result (class tested)	Subsequent contrast administration	Class concordance	New HSR
RCM-01	65/F	ICM (iohexol)	IHR, GI	18	− (ICM, culprit)	GBCA, PM−	Discordant	−
RCM-02	53/F	ICM, NS	IHR, GII	92	NA	Imaging without contrast	NA	NA
RCM-03	51/F	ICM, NS	IHR, GIII	15	NA	Imaging without contrast	NA	NA
RCM-04	66/F	ICM, NS	IHR, GII	483	− (ICM)	ICM, PM+	Concordant	−
RCM-05	39/F	GBCA, NS	IHR, GII	61	+ (GBCA)	GBCA, PM+	Concordant	−
RCM-06	61/F	ICM, NS	IHR, GII	360	− (ICM)	ICM, PM−	Concordant	−
RCM-07	65/F	ICM (iohexol)	NIHR	1	− (ICM)	No imaging	NA	NA
RCM-08	81/F	ICM, NS	IHR, GII	3	NA	No imaging	NA	NA
RCM-09	63/M	ICM (iohexol)	IHR, GIII	1	NA	ICM, PM+	Untested	+
RCM-10	51/F	ICM (iohexol)	IHR, GIII	60	− (GBCA)	ICM, PM+	Discordant	+
RCM-11	59/F	ICM, NS	IHR, GIII	4	− (ICM+GBCA)	No imaging	NA	NA
RCM-12	36/F	ICM (iohexol)	IHR, GIII	3	− (ICM, culprit)	Imaging without contrast	NA	NA
RCM-13	50/M	GBCA, NS	NIHR	1	− (GBCA)	Imaging without contrast	NA	NA
RCM-14	58/F	GBCA, NS	NIHR	240	− (GBCA)	GBCA, PM−	Concordant	−
RCM-15	34/F	ICM (iohexol)	IHR, GIII	3	− (GBCA)	ICM, PM+	Discordant	+
RCM-16	51/F	GBCA, NS	IHR, GII	64	− (ICM)	Not contactable	NA	NA
RCM-17	46/F	ICM (iohexol)	IHR, GIII	4	− (GBCA)	GBCA, PM+	Concordant	−
RCM-18	73/M	ICM, NS	IHR, GII	516	− (ICM)	ICM, PM+	Concordant	−
RCM-19	63/F	ICM, NS	IHR, GIII	184	− (ICM+GBCA)	Imaging without contrast	NA	NA
RCM-20	60/F	ICM (iohexol)	IHR, GIII	2	− (ICM+GBCA, culprit)	Imaging without contrast	NA	NA
RCM-21	58/M	GBCA (gadobutrol)	NIHR	23	− (GBCA, culprit)	No imaging	NA	NA
RCM-22	76/F	ICM, NS	IHR, GI	240	− (ICM)	ICM, PM+	Concordant	−
RCM-23	69/F	GBCA, NS	IHR, GII	60	− (ICM+GBCA)	No imaging	NA	NA
RCM-24	63/M	GBCA, NS	IHR, GI	216	− (GBCA)	GBCA, PM+	Concordant	−
RCM-25	63/M	ICM (iohexol)	NIHR	202	NA	No imaging	NA	NA
RCM-26	45/F	ICM (iopromide)	IHR, GII	66	NA	GBCA, PM−	Untested	−
RCM-27	53/F	ICM, NS	IHR, GIII	246	NA	Imaging without contrast	NA	NA
RCM-28	51/F	ICM, NS	IHR, GIII	6	NA	GBCA, PM−	Untested	−

Patient	Age/sex	Culprit agent	Index reaction	Time to allergy evaluation, months	Skin test result (class tested)	Subsequent contrast administration	Class concordance	New HSR
RCM-29	63/M	ICM, NS	IHR, GII	1	NA	No imaging	NA	NA

Time to allergy evaluation is the number of months between the index reaction and the allergy evaluation; subsequent contrast administration refers to contrast-enhanced imaging performed after the allergy evaluation during routine clinical care. Skin test result is followed by the contrast class tested; culprit marks the four patients whose testing panel included the culprit agent (diagnostic strategy). Class concordance compares the class tested with the class administered: concordant, the class administered had been tested; discordant, only the other class had been tested; untested, no skin testing was done. Abbreviations: G, Brown grade; GBCA, gadolinium-based contrast agent; HSR, hypersensitivity reaction; ICM, iodinated contrast media; IHR, immediate hypersensitivity reaction; NA, not applicable; NIHR, non-immediate hypersensitivity reaction; NS, not specified; PM+, premedication reported; PM−, premedication not reported; +, positive/present; −, negative/absent.

Table S2. STROBE Statement—checklist of items that should be included in reports of cohort studies, with the location of each item in the manuscript.

Item	No.	Recommendation	Reported in (section / element)
**Title and abstract**	1	(a) Indicate the study's design with a commonly used term in the title or the abstract	Title; Abstract (Methods: "single-centre retrospective cohort")
		(b) Provide in the abstract an informative and balanced summary of what was done and what was found	Abstract
**Introduction**			
**Background/rationale**	2	Explain the scientific background and rationale for the investigation being reported	Section 1, paragraphs 1–7
**Objectives**	3	State specific objectives, including any prespecified hypotheses	Section 1, final paragraph; Abstract (Background/Objectives)
**Methods**			
**Study design**	4	Present key elements of study design early in the paper	Section 2.1
**Setting**	5	Describe the setting, locations, and relevant dates, including periods of recruitment, exposure, follow-up, and data collection	Sections 2.1, 2.2 and 2.5 (evaluation January 2019–May 2025; telephone interview July 2026)
**Participants**	6	(a) Give the eligibility criteria, and the sources and methods of selection of participants; describe methods of follow-up	Section 2.2 (147 referred; 98 without a compatible history; 12 did not attend the evaluation; 8 insufficient records; 29 included); Section 2.5 (retrospective ascertainment by structured telephone interview and electronic records); Figure 1a
		(b) For matched studies, give matching criteria and number of exposed and unexposed	Not applicable
**Variables**	7	Clearly define all outcomes, exposures, predictors, potential confounders, and effect modifiers; give diagnostic criteria, if applicable	Sections 2.3, 2.4 and 2.6; Section 2.7 (index severity and reaction-to-evaluation interval as potential confounders)
**Data sources/measurement**	8	For each variable of interest, give sources of data and details of methods of assessment (measurement); describe comparability of assessment methods if there is more than one group	Sections 2.3–2.5 (clinical records, referral reports, national electronic health records, structured telephone interview)
**Bias**	9	Describe any efforts to address potential sources of bias	Section 2.5 (verification of contrast administration in electronic records); Section 2.7 (Holm adjustment; Firth penalised regression); Section 4 (limitations: selection and indication bias, recall)
**Study size**	10	Explain how the study size was arrived at	Section 2.7 (no a priori sample size calculation; all eligible patients in the study period)
**Quantitative variables**	11	Explain how quantitative variables were handled in the analyses; if applicable, describe which groupings were chosen and why	Section 2.7 (Shapiro–Wilk; $\log_2$-transformed interval; Grade III vs. Grade I–II)
**Statistical methods**	12	(a) Describe all statistical methods, including those used to control for confounding	Section 2.7 (Fisher's exact test; Mann–Whitney U test with exact permutation p values accounting for ties; Holm adjustment of unrounded p values for 6 exploratory comparisons; Firth penalised logistic regression adjusted for index severity)
		(b) Describe any methods used to examine subgroups and interactions	Section 2.7 (immediate-reaction subgroup; ICM subgroup)

Item	No.	Recommendation	Reported in (section / element)
		(c) Explain how missing data were addressed	Sections 2.6 and 2.7 (denominators reported; patients with unknown outcomes not included in the no-reaction group; tryptase analysis restricted to patients with measurements)
		(d) If applicable, explain how loss to follow-up was addressed	Section 2.5 (not contactable after three calls; classified as unknown outcome); Section 3.4 (28/29 with post-evaluation information); Figure 2
		(e) Describe any sensitivity analyses	Section 2.7 (Firth sensitivity model additionally including the log_2-transformed reaction-to-evaluation interval); Section 3.4
**Results**			
**Participants**	13	(a) Report numbers of individuals at each stage of study; (b) give reasons for non-participation at each stage; (c) consider use of a flow diagram	Section 2.2 and Figure 1a (147 → 29 with reasons for exclusion); Section 3.4 and Figure 2 (28 with outcome information, 21 imaged, 14 given contrast, 7 without contrast, 7 not imaged, 1 not contactable)
**Descriptive data**	14	(a) Give characteristics of study participants and information on exposures and potential confounders; (b) indicate number of participants with missing data for each variable of interest; (c) summarise follow-up time	Sections 3.1–3.2 and Tables 1–2 (characteristics; n given for IgE, eosinophils and tryptase; culprit agent not documented by name in 18); Section 3.4 (median 42 months from allergy evaluation to interview); Table S1
**Outcome data**	15	Report numbers of outcome events or summary measures over time	Sections 3.3–3.5 (skin test positivity 1/20; new reactions 3/14; only 1 death in the cohort); Table 3; Figures 2 and 3; Table S1
**Main results**	16	(a) Give unadjusted estimates and, if applicable, confounder-adjusted estimates and their precision; (b) report category boundaries when continuous variables were categorised; (c) if relevant, consider translating estimates of relative risk into absolute risk	Sections 3.2, 3.4 and 3.5 (proportions with Wilson 95% CI; unadjusted and Holm-adjusted p values; Firth-adjusted odds ratios with 95% profile penalised-likelihood CI; interval modelled as log_2 months)
**Other analyses**	17	Report other analyses done—e.g., analyses of subgroups and interactions, and sensitivity analyses	Section 3.4 (ICM subgroup; distribution of the 13 patients in the Firth models; sensitivity model); Section 3.5 (baseline tryptase, exploratory)
**Discussion**			
**Key results**	18	Summarise key results with reference to study objectives	Section 4, paragraph 1; Section 5
**Limitations**	19	Discuss limitations of the study, taking into account sources of potential bias or imprecision; discuss both direction and magnitude of any potential bias	Section 4, limitations paragraphs (retrospective design; single interview; non-standardised premedication; selection and indication bias; small sample; GBCA test concentrations; unknown older preparations)
**Interpretation**	20	Give a cautious overall interpretation of results considering objectives, limitations, multiplicity of analyses, results from similar studies, and other relevant evidence	Section 4; Section 5
**Generalisability**	21	Discuss the generalisability (external validity) of the study results	Section 4 (single-centre tertiary setting; selection and indication bias)
**Other information**			
**Funding**	22	Give the source of funding and the role of the funders for the present study	Funding statement (no external funding)

STROBE, Strengthening the Reporting of Observational Studies in Epidemiology (von Elm et al., Lancet 2007;370:1453–1457).
